# Recurrent Primary Peritoneal Psammocarcinoma: A Case Report on a Diagnostic Challenge in Cytology

**DOI:** 10.7759/cureus.41964

**Published:** 2023-07-16

**Authors:** FNU Sapna, Antonio Cajigas, Raja Sandeep Perkash, Xiaohua Qi, Shweta Gera

**Affiliations:** 1 Pathology, Albert Einstein College of Medicine, Bronx, USA; 2 Neurological Surgery, Albert Einstein College of Medicine, Bronx, USA

**Keywords:** recurrence, cytology diagnosis., low grade primary peritoneal serous carcinoma, metastasis, primary peritoneal psammocarcinoma

## Abstract

Psammocarcinoma (PCa) is a rare variant of low-grade papillary serous carcinoma that can arise from the peritoneal as well as ovarian surfaces. When this tumor involves the extra-ovarian peritoneum significantly and the ovarian surface minimally or not at all, it is considered of peritoneal origin. PCa has a recurrent indolent clinical course. It is challenging to diagnose peritoneal PCa, particularly on cytological smears because of the bland cellular features of neoplastic cells. We report a case of recurrent metastatic primary peritoneal PCa in a 71-year-old female of Ashkenazi Jewish ancestry diagnosed on cytology of ascitic and cystic fluid.

## Introduction

Psammocarcinoma (PCa) is a rare subtype of low-grade papillary serous carcinoma of the peritoneum and ovary. This rare epithelial tumor was first described by Kettle in 1916. Peritoneal PCa is rarer than ovarian PCa. The number of reported cases of peritoneal PCa in English literature is only around 30 cases. Gilks et al., in 1990, proposed a criterion for its diagnosis on histology, consisting of four distinct histologic patterns, including (a) ovarian stromal invasion, vascular invasion or an extra-ovarian localization, and invasion of intraperitoneal viscera, (b) mild cytological atypia, (c) few cellular nests with no more than 15 cells in number; however, no areas of solid epithelial growth, and (d) psammoma bodies replacing at least 75% of the papillae [1]. There is limited information available about its clinical behavior, prognosis, and treatment. After raising awareness of this entity, it was noted that it has identical clinical characteristics to serous borderline tumors of the peritoneum and ovary, having a long clinical course and a good prognosis [2,3]. In 1994, Chen et al. expanded Gilks' first criterion that either infiltration of the intraperitoneal viscera or invasive growth pattern in the peritoneum should be reserved for diagnosis of primary peritoneal PCa [4]. Management typically involves surgery and chemotherapy. It has been noted that this entity can recur even after optimal treatment. We report a unique case of recurrent primary peritoneal psammocarcinoma with its recurrence diagnosed on cytology, which can be extremely challenging because of mild cellular atypia of malignant epithelial cells.

## Case presentation

**Figure 1:**
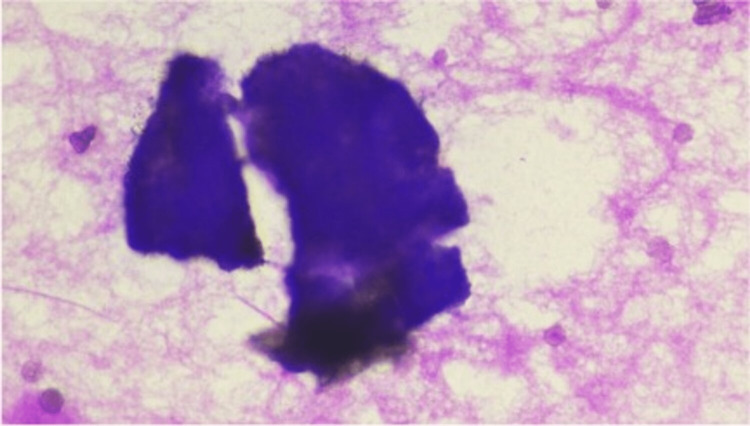
Diff Quik stain on a smear from ascitic fluid (40x) Degenerated cells with calcifications

**Figure 2:**
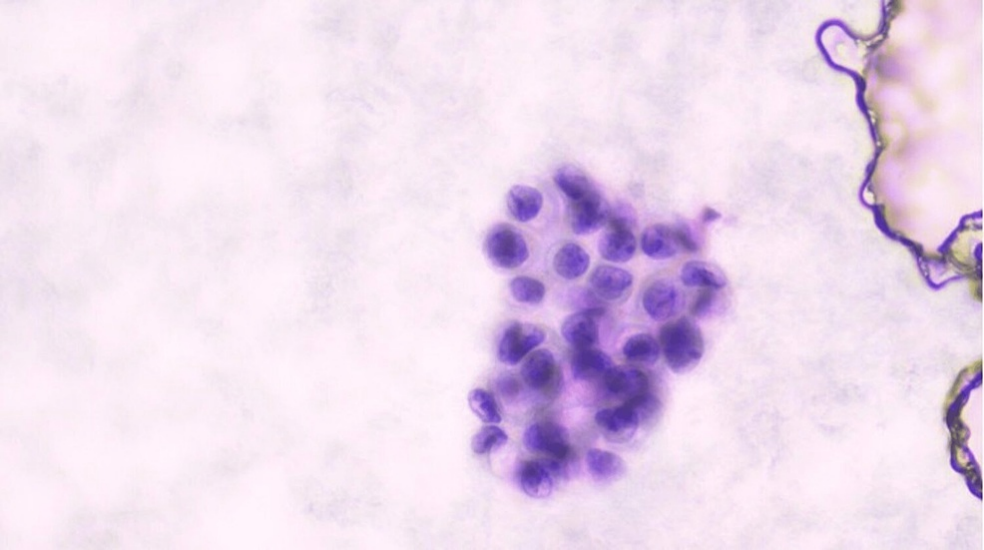
Papanicolaou stain on a smear from the ascitic fluid (40x) Medium-sized cells with mild atypia in three-dimensional round configurations

A 71-year-old Ashkenazi Jewish female presented with abdominal distension and nausea. Laboratory investigations revealed raised serum level of cancer antigen-125 (CA-125) of 54,326.4 U/ml (reference range: <35U/ml). Abdominal computed tomography (CT) scan revealed a cystic lesion in the left lateral abdomen in the gastrohepatic ligament measuring approximately 3.7 cm (about 1.46 inch) and showed loculated ascites in the mid-abdomen surrounding loops of the small bowel. These findings were also noted in previous imaging. At the age of 51 years, she was diagnosed with primary peritoneal PCa on the surgical specimen of hysterectomy, bilateral salpingo-oophorectomy (BSO), omentectomy, and local lymph node resection, which was performed because of the finding of omental mass on imaging that was mostly involving the peritoneum and was confined only to the surface of the left ovary. After surgery, she received chemotherapy, including cisplatin and cyclophosphamide, and was disease-free for five years. She developed ascites at the age of 56 years, which was drained, and ascitic fluid cytology showed degenerated cells with calcifications on Diff-Quik stain (Figure 1) and medium-sized cells with mild atypia in three-dimensional round configurations on Papanicolaou stain (Figure 2).

**Figure 3:**
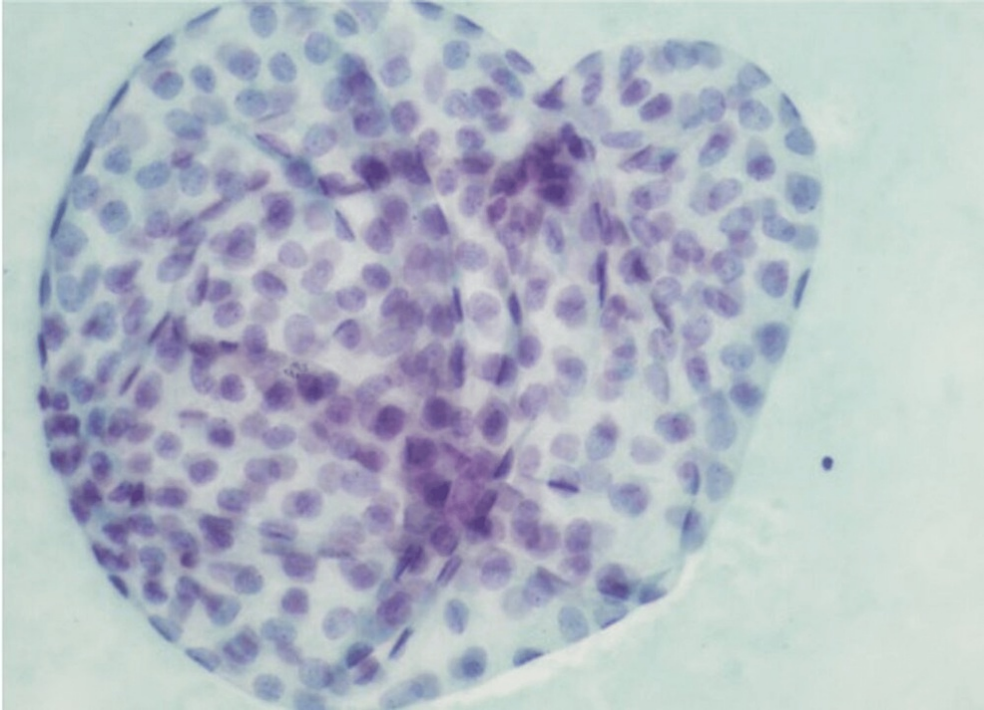
Papanicolaou stain on a smear from the cystic fluid in the gastrohepatic ligament (40x)

**Figure 4:**
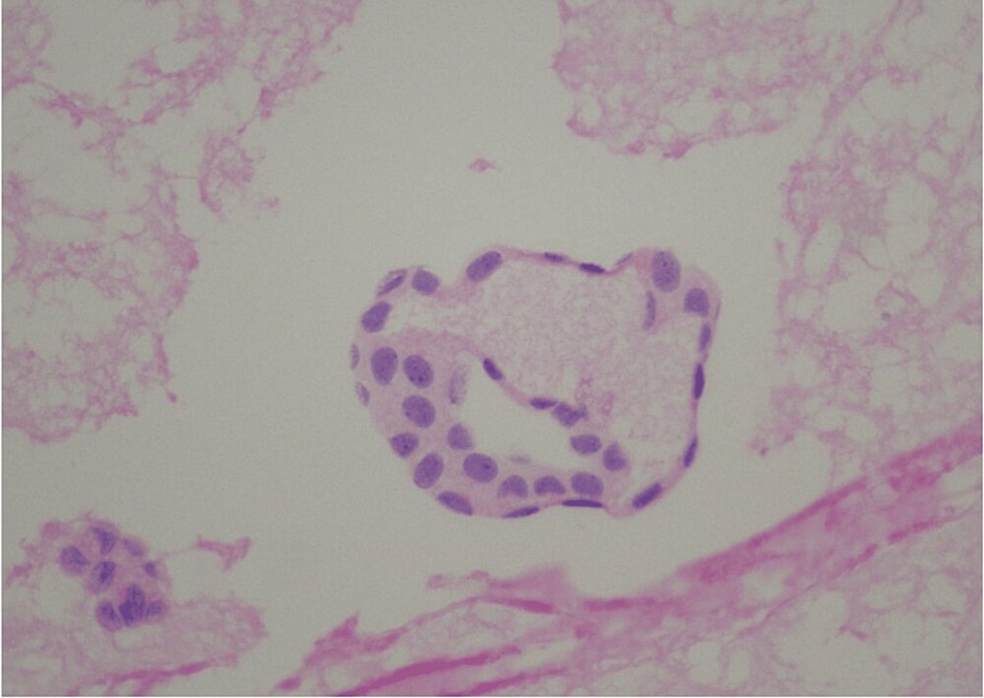
Hematoxylin and eosin (H&E) stain on a smear from the cystic fluid (40x)

The cellular features compared with a previous tumor found on histology were similar, having very mild atypia, so it was suggested to be a recurrent metastatic primary peritoneal PCa. She was again given a course of chemotherapy with doxorubicin. After 15 years, at the age of 71 years, she presented with abdominal distension and nausea having imaging findings described earlier. She underwent drainage of the cystic lesion and peritoneal ascites. Cytology smears from both sites showed similar three-dimensional mildly atypical cell clusters. Papanicolaou stain on smear of cystic fluid in a gastrohepatic ligament (Figure 3), and hematoxylin and eosin (H&E) stained slide revealed cells with similar morphology (Figure 4).

**Figure 5:**
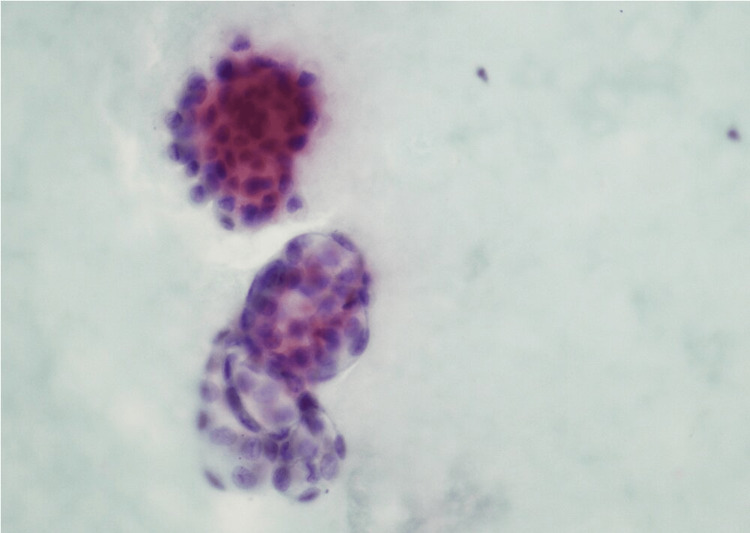
Papanicolaou stain on the smear of ascitic fluid (40x)

On Papanicolaou stain, the ascitic fluid smear also showed similar three-dimensional, mildly atypical cell clusters with amorphous material within the center (Figure 5).

Based on the history of tumor recurrence, imaging, cytologic and histologic features, the diagnosis of recurrent metastatic primary peritoneal PCa was favored.

## Discussion

Psammocarcinoma (PCa) is an extremely rare variant of low-grade papillary serous carcinoma of the peritoneum and ovary associated with extensive psammoma bodies [4-6]. The main factor in the development of psammoma bodies is the buildup of hydroxyapatite in dividing cells, which results in dystrophic calcifications [4,6,7]. Both benign and malignant disorders of the peritoneum, gynecologic tract, as well as other organs like the thyroid, have been associated with psammoma bodies [8]. Gilks et al., in 1990, proposed a criterion for its diagnosis on histology, consisting of four distinct histologic patterns, including (a) ovarian stromal invasion, vascular invasion or an extra-ovarian localization, and invasion of intraperitoneal viscera, (b) mild cytological atypia, (c) few cellular nests with no more than 15 cells in number; however, no areas of solid epithelial growth, and (d) psammoma bodies replacing at least 75% of the papillae [1,4,9]. In 1994, Chen et al. expanded Gilks' first criterion that either infiltration of the intraperitoneal viscera or invasive growth pattern in the peritoneum should be reserved for diagnosis of primary peritoneal PCa [4,10]. Both ovarian and peritoneal PCa have similar demographics and histologic characteristics while the distinction between the two is dependent upon the extent of involvement of the two sites. Ovarian PCa has ovarian stromal invasion [3,10] while in peritoneal PCa, there is extensive peritoneal involvement with minimal ovarian involvement limited only to the ovarian surface. Our patient did not have ovarian stromal invasion but had extensive involvement of the peritoneum by a tumor on histology.

The cytologic characteristics of peritoneal PCa were additionally investigated for the first time by Chen et al. on peritoneal washings. He clarified that on smears, the diagnosis of peritoneal PCa can only be presumed from cytologic features alone because the invasive pattern cannot be determined from the cytologic specimens, thus it is extremely difficult to diagnose peritoneal PCa on cytology because of bland cellular features and mild atypia of neoplastic cells that can easily be missed. It can be difficult to determine whether the PCa is ovarian or peritoneal in origin. Only a histologic examination in such circumstances can distinguish peritoneal PCa from ovarian PCa. The most significant characteristic of ovarian PCa is ovarian stromal invasion, which is not seen in peritoneal PCa [10]. Based on Gilks and Chen’s criteria, our case met the criteria of primary peritoneal PCa on histology. The development of malignant ascites with similar atypical cells favored its recurrence.

Differential diagnoses included mesothelioma, usual serous adenocarcinoma, and leiomyomatosis peritonealis disseminata. To rule out mesothelioma, we performed immunohistochemistry, which should show positivity for CK5/6 and D2-40, but these markers were negative in our patient. Usual serous adenocarcinoma can easily be ruled out based on high-grade cytological features in neoplastic cells. Leiomyomatosis peritonealis disseminata is a rare entity that only affects young women with a history of myomectomy [11].

Patients present with nonspecific physical signs and symptoms of growing abdominal girth, discomfort, or sometimes nausea [6]. Regarding serum markers, it is unclear how elevated CA-125 values are related to the progression of the disease. In certain circumstances, it can result in increased serum CA-125 levels [5,6]. An elevated serum CA-125 level may indicate a propensity for aggressive behavior [12]. Our patient also had very high levels of CA-125 at presentation while having an indolent course.

Due to the rarity of primary peritoneal PCa, its care is not standardized [4-6,13,14]. However, many authors advise optimal surgical tumor debulking and adjuvant chemotherapy [3,5,15,16]. Maximum surgical debulking, including bilateral salpingo-oophorectomy, total abdominal hysterectomy, and omentectomy is the cornerstone of the treatment [5]. Only young women diagnosed with PCa are candidates for conservative surgery [12]. According to Munkarah et al., there are no definitive recommendations for postoperative chemotherapy [13]. There are data regarding the effectiveness of hormonal therapy with tamoxifen in patients with recurrent disease. A case of recurrent primary peritoneal PCa that completely responded to tamoxifen therapy was described by Molpus et al. [6]. Weir et al. also gave tamoxifen to those patients with recurring illnesses [12]. According to previous literature, PCa has a better prognosis than typical serous carcinomas, but it can still have a clinically aggressive, recurring, and metastatic course that needs systemic therapy [17]. In our patient, optimal debulking was performed, followed by postoperative adjuvant systemic chemotherapy. This treatment strategy was well tolerated by our patient without any side effects, but it could not prevent relapse, thus more treatment options should be elucidated that can prevent recurrences and metastases. 

## Conclusions

Primary peritoneal psammocarcinoma (PCa) is an extremely rare variant of low-grade primary peritoneal serous cancer. Low-grade cytology, psammoma bodies, and invasiveness are distinguishing hallmarks on histologic examination, while on cytological smears, diagnosing PCa is quite difficult. Hence, one requires a strong index of suspicion while looking at smears. Given its bland appearance and lack of invasiveness on smears, it can be readily misinterpreted if the history, clinical presentation, and radiological findings are not carefully investigated. This uncommon entity has a good overall prognosis and an indolent clinical course, but multiple recurrences can occur despite chemotherapy as in our case. To minimize the recurrence rate, additional studies are necessary to find the optimum course of treatment. Adding more cases of PCa in the literature will help understand its clinical behavior and outcome more in-depth so that effective treatment approaches can be taken place, as there is no standardized treatment for this entity because of its rarity.
